# Measuring electro-mechanical properties of thin films on polymer substrates

**DOI:** 10.1016/j.mee.2014.08.002

**Published:** 2015-04-02

**Authors:** Megan J. Cordill, O. Glushko, J. Kreith, V.M. Marx, C. Kirchlechner

**Affiliations:** a Erich Schmid Institute of Materials Science, Austrian Academy of Sciences, Jahnstrasse 12, 8700 Leoben, Austria; b Department of Material Physics, Montanuniversität Leoben, Jahnstrasse 12, 8700 Leoben, Austria; c Max-Planck-Institut für Eisenforschung GmbH, Max-Planck-Str. 1, 40237 Düsseldorf, Germany

**Keywords:** AFM, Fragmentation, Thin films, Flexible substrates, Resistance, In-situ

## Abstract

In order to advance flexible electronic technologies it is important to study the electrical properties of thin metal films on polymer substrates under mechanical load. At the same time, the observation of film deformation and fracture as well as the stresses that are present in the films during straining are also crucial to investigate. To address both the electromechanical and deformation behavior of metal films supported by polymer substrates, in-situ 4 point probe resistance measurements were performed with in-situ atomic force microscopy imaging of the film surface during straining. The 4 point probe resistance measurements allow for the examination of the changes in resistance with strain, while the surface imaging permits the visualization of localized thinning and crack formation. Furthermore, in-situ synchrotron tensile tests provide information about the stresses in the film and show the yield stress where the deformation initiates and the relaxation of the film during imaging. A thin 200 nm Cu film on 23 μm thick PET substrate will be used to illustrate the combined techniques. The combination of electrical measurements, surface imaging, and stress measurements allow for a better understanding of electromechanical behavior needed for the improvement and future success of flexible electronic devices.

## Highlights

• Electromechanical properties of metal films are studied with in-situ AFM-4PP tensile straining.
• AFM characterizes the surface damage and 4PP measures the resistance under tensile strain.
• The difference between local film necking and through thickness cracks can be made.
• It will be shown when the strain is removed, cracks bridge and the resistance decreases.

## Graphical abstract

**Figure:**
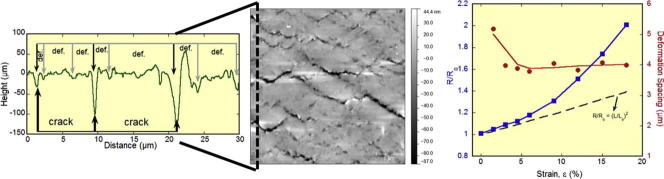

## 1 Introduction

Advances in fabrication and design are making flexible electronic devices and sensors more available. From flexible solar cells [1–2], wearable sensors [3–5], and foldable displays [6,7], these devices will soon become a reality. The special combination of stiff films and islands connected by deformable metal lines and supported by a compliant polymer substrate is important for flexible device design and reliability. It is essential that these material systems have consistent electrical and mechanical behavior over a wide range of loading conditions (stretching, bending, rolling, twisting, etc.). However, measuring the combined electro-mechanical properties and investigating the failure mechanisms is often difficult, requiring the development or advancement of new techniques.

**Fig. 1:**
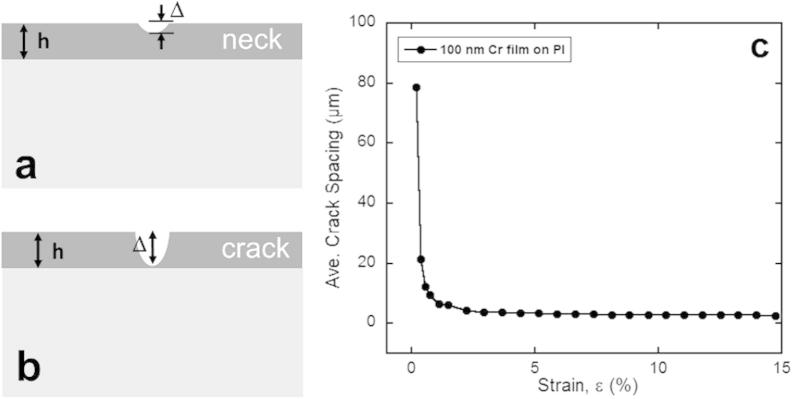
Schematic diagram of (a) local necking and (b) through thickness channel cracks found during uni-axial tensile straining of ductile films. (c) Example of the crack spacing evolution as a function of applied tensile strain for a 100 nm Cr film on PI.

In order to study the mechanical behavior of metal films on compliant polymer substrates, fragmentation testing is often employed [8–12]. During fragmentation testing, the film-substrate couple is strained under uni-axial tension and observed with light microscopy (LM) or scanning electron microscopy (SEM). Brittle metals or ceramic films fracture, forming through thickness cracks (channel cracks) at low strain perpendicular to the straining direction. On the other hand, ductile metal films will first deform locally in the form of necks at low strains (Fig. 1a) and with increased strain through thickness cracks (TTC) can evolve (Fig. 1b). Fragmentation testing is best performed in-situ with LM or SEM so that the strain when the first crack forms can be observed. The initial fracture strain of the film, also known as the crack onset strain, can then be used to determine the interfacial fracture shear stress with knowledge of the crack spacing at saturation, *λ*, film thickness, *h*, and the fracture stress, *σ*_f_ = *E*_film_*ε*_f_, where *ε*_f_ is the fracture strain, using the shear lag model [8,13,14]. In-situ fragmentation testing with LM or SEM allows for the crack spacing evolution to be observed as a function of applied strain (Fig. 1c). Under tensile straining conditions, a brittle film will initially fracture at very low strains (<1%) and then with further strain continue to form cracks until the saturation crack spacing is reached. After the saturation spacing has been reached, cracks can no longer form between existing crack fragments and the film could delaminate via buckling.

LMs and SEMs are very good imaging techniques for brittle films, however, the same methods are more difficult to use on ductile films like Cu and Au because the SEM is unsuited to resolve small changes in surface height, especially at large working distances required to accommodate in-situ straining stages. For fragmentation testing of ductile films, atomic force microscopy (AFM) is an approach that allows for the recognition of TTC and localized necking at the film surface [15–18]. With the ability to distinguish between a TTC and a neck, the spacing of TTC and necks can be quantified as a function of strain. In-situ AFM fragmentation testing has also been utilized on brittle films to reveal bending the crack fragments under strain [14,19] which could not be detected without the height resolution of the AFM.

**Fig. 2:**
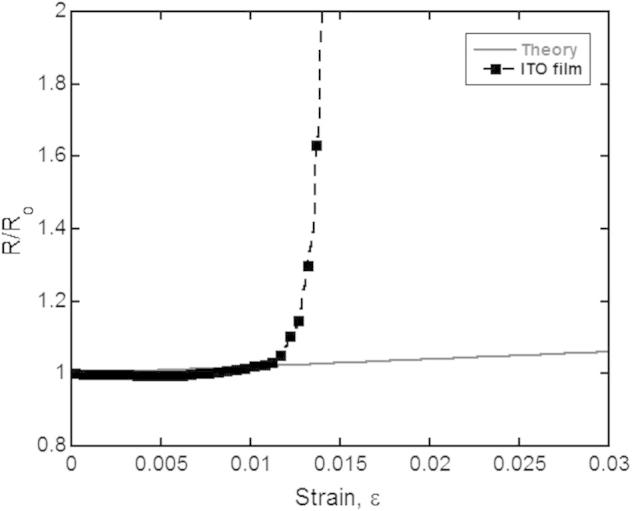
When indium tin oxide (ITO) is strained in tension with in-situ 4PP resistance measurements, the film fractures at low strains and the relative resistance ratio (*R*/*R*_o_) dramatically increases away from the predicted theory (straight line).

Another in-situ fragmentation test measures the resistance of the film using the 4 point probe (4PP) geometry [20–23]. The advantage of this in-situ technique is that the exact fracture strain can be determined for brittle films. The fracture strain of the film can be determined when the resistance ratio deviates from the theoretical resistance ratio (Fig. 2). To date, in-situ 4PP fragmentation testing has been utilized on both brittle and ductile films on polymer substrates, with more advanced work on ductile films. These advances with in-situ 4PP have occurred on ductile films because the resistance gradually increases with applied strain rather than abruptly increasing to infinity. The theoretical background was also developed using ductile Cu films on polyimide [20,22,24]. For the ideal situation of a homogeneous, uniform and stress-free film the initial resistance, *R*_o_, before straining is described as

(1) $$R_{0}=\rho \frac{L_{0}}{A_{0}}$$

where *ρ* is the resistivity, *L*_o_ is the initial distance between the contacts, and *A*_o_ is the initial cross-section of the film. It should be noted that *L*_o_ is assumed to be equal to the initial gauge length. During straining the instantaneous resistance is described by

(2) $$R=\rho \frac{L}{A}$$

where *L *= *L*_o_ + Δ*L* is the instantaneous gauge length with the strain is defined as *ε *= Δ*L*/*L*_o_ and *A* is the instantaneous cross-section of the film. As the film is deformed plastically under tension, it is assumed that the volume of the film remains constant which gives rise to the very simple relation between the relative resistance (*R*/*R*_o_) and the relative elongation (*L*/*L*_o_)

(3) $$\frac{R}{R_{0}}=\left(\frac{L}{L_{0}}\right)\equiv {\left(1+∊\right)}^{2}$$

When the film displays significant structural modification through the formation of cracks, Eq. (3) is no longer valid and the resistance is difficult to describe by an analytical formula. It should also be noted that although many researchers utilize in-situ 4PP fragmentation testing, the direct correspondence between the crack spacing and resistance for ductile films has not been demonstrated.

In this study, in-situ 4PP and in-situ AFM fragmentation methods are combined to investigate the role of film mechanical deformation on the electrical behavior. Localized necking of the film is the first sign of yield but it is unknown how the initial deformation affects the electrical behavior. By combining the two methods, a direct measurement and correlation of the induced mechanical deformation and the electrical properties as a consequence of the deformation can be measured. From the experiments, new insights into electromechanical behavior of metal films on polymer substrates can be uncovered.

## 2 Materials and methods

Ductile copper films were sputter deposited onto 23 μm thick polyethylene terephthalate (PET) substrates using an industrial method. In order to improve the adhesion of the 200 nm Cu to the PET substrates, a 5 nm Cr layer was deposited before the Cu film. The Cu films had an average grain size of about 100 nm which was measured using ion channeling contrast images.

Uni-axial tensile straining was performed on rectangular samples with approximate dimensions of 8 mm × 35 mm. For the combined electro-mechanical behavior, the samples were strained under a Dimension 3100 AFM utilizing a custom built small-scale straining stage which is screw-driven [15,16]. The AFM was used to image the Cu surface and to quantify the amount of mechanical damage as a function of strain. It should be noted that the custom built straining stage can only be used in displacement control, thus only the strain was determined. At every straining step, a 30 μm × 30 μm image was obtained of the same area using tapping mode. The AFM stage was adapted so that the straining stage could be fastened tightly to it. This adaption made it easy to always find the same area on the sample and no additional markers on the sample were needed. In order to capture the electrical behavior, four point probes were incorporated into the grips of the straining stage to measure the resistance of the film during straining [24]. The electrical resistance was measured with a Keithley 2400 source meter and the data recorded using a PYTHON script. The measurement combination of the electrical resistance and mechanically induced damage as a function of strain allowed for a direct comparison of both behaviors at once.

An experiment is performed in the following manner. A sample is loaded and the original gauge length and resistance and an image in the unstrained position are recorded. Then the sample is strained in small increments (approximately 1.5–3% steps) and the procedure repeated (gauge length and resistance measured, AFM image made) until the desired maximum strain. Strain increments of 1.5% were used in the early stages of straining in order to better capture the initial surface deformation and cracking. Once these events had been observed (after 6% strain), the straining increment was increased to 3%. In the final step, the strain was carefully reduced so that the sample was in a flat, unloaded state and another set of measurements was performed. The electrical resistance data was then normalized by the original resistance, *R*_o_, so it could be plotted as a function of strain as in Eq. (3).

**Fig. 3:**
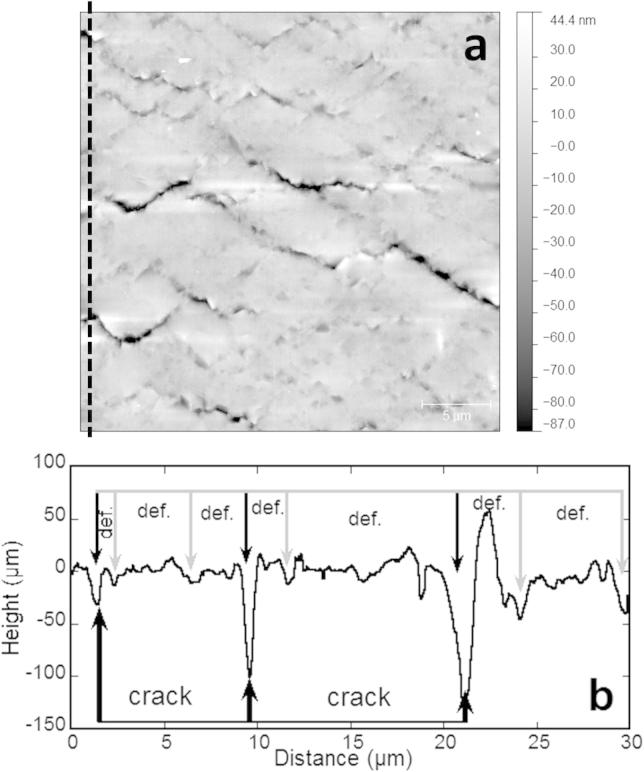
(a) AFM height image of 200 nm Cu film on PET strained in-situ under the AFM. (b) Surface profiles extracted from the AFM image (dashed line in a) with deformation (def.) and cracks indicated.

The AFM images were analyzed following the process reported by Cordill and Marx [15,16]. For every image (straining step), three surface profiles were taken at the left and right edges of the image as well as in the middle of the image approximately at the same place for each step (Fig. 3a). Necks and TTCs were identified in the surface profiles as places where the surface profile dramatically decreases (Fig. 3b). At these positions, the average depth, Δ, of the neck or crack was calculated using the surface profile data 250 nm on both sides of the lowest point. With the defined criteria, a neck was classified as having a Δ*/h < *0.15 and a TTC was classified as Δ*/h* > 0.15, where *h* is the film thickness. From the determination of a neck or TTC, the average deformation spacing and crack spacing were determined.

As a comparison, evolution of the stress of the Cu film on PET was also measured in-situ using the X-ray based sin^2^ *ψ* method. This type of in-situ fragmentation procedure allows for the direct measurement of the lattice strains. The in-situ sin^2^ *ψ* measurements were conducted at the KMC-II beam line at the Berliner Elektronenspeicherring–Gesellschaft für Synchrotronstrahlung (BESSY II) in Berlin, Germany [25]. Using a Vantec 2000 (Brucker AXS, Germany) and a 5 s exposure time, the monochromatic 7 keV beam (diameter of 300 μm) was used to measure the Cu (1 1 1) reflection at four different *ψ* angles. The samples were continuously strained with an Anton Paar TS600® straining stage at a displacement rate of 2 μm/s to 15% with a 5 min hold before unloading to zero force at the same displacement rate. The lattice strains were then used to derive the stresses applying the X-ray elastic constants (½ S_2_) of an untextured copper film. The film stresses were correlated to the compound stress-strain curves of the film and substrate measured with the calibrated load cell and strain gauge of the TS600.

## 3 Results and discussion

**Fig. 4:**
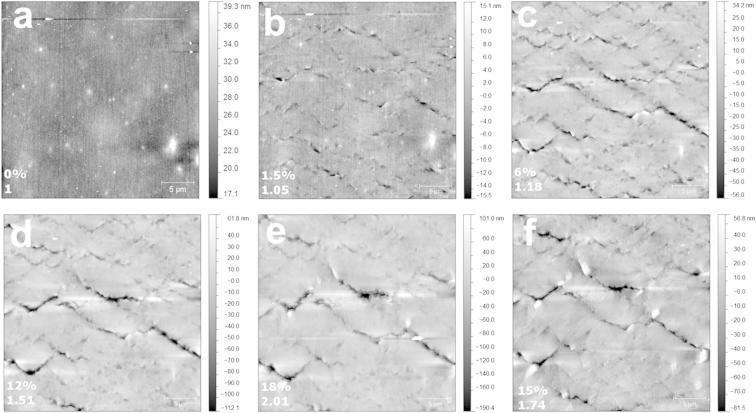
Example of the in-situ AFM-4PP experiment. (a) Unstrained condition, (b) 1.5% strain, first detection of localized necking on the film surface, (c) 6% strain after TTC have formed, (d) 12% strain, the start of the saturation crack spacing regime, (e) maximum strain of 18%, 200% increase of resistance, (f) unloaded film at 15% strain and the relative resistance ratio is lower than at the maximum strain (1.74). In each image, the two numbers in the bottom left corners are the percent strain and relative resistance ratio, respectively.

**Fig. 5:**
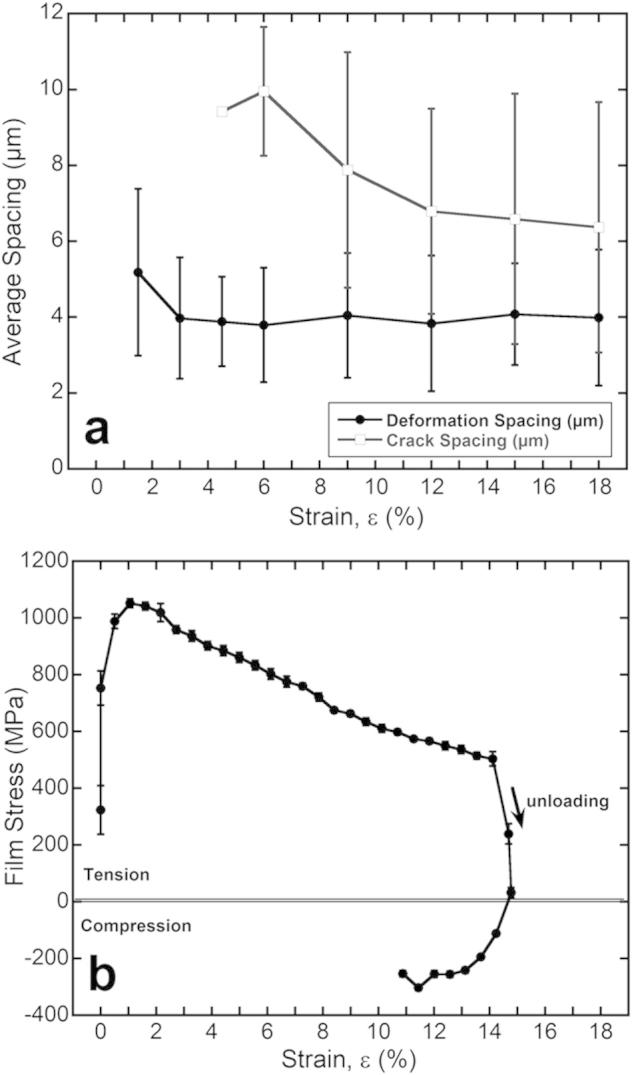
(a) In-situ AFM allows for the separate quantification of the deformation spacing (necks and TTC) and crack spacing (TTC only). The deformation spacing reaches saturation at 6% strain which corresponds to TTC formation. The TTC saturation occurs at approximately 12% strain. (b) In-situ XRD stress measurements of the same film illustrating that the maximum stress is reached at approximately 1% strain and upon unloading, the film retains a high compressive stress.

The 200 nm Cu films on the 23 μm thick PET substrate were strained in-situ under the AFM and with 4PP resistance measurements. From the images obtained during the straining steps, the deformation spacing and crack spacing was determined using the Δ*/h* criteria described in the previous section. A series of AFM images are shown in Fig. 4. As expected, at low strains (1.5%) only local surface deformation was observed and the deformation spacing consisted only of necking areas (Fig. 4b). With increased strain, cracks developed at a few of the initial necking sites. The first through thickness crack formed between 4.5% strain and 6% strain (Fig. 4c and d). At approximately 6% strain the deformation spacing reached its saturation value and the TTCs continued to form, reaching saturation at about 15% strain before the experiment was stopped at a strain of 18% (Fig. 4e). The tension was then released to an unloaded strain of 15% (Fig. 4f). It should be noted that the straining device does not measure the load during straining, therefore, the unloaded strain was determined when the sample became flat between the grips when the tension was slowly released but before the sample buckled between the grips due to sample elongation. The crack and deformation spacing evolution is shown in Fig. 5a. The error bars are the standard deviation of the crack and deformation spacing measurements from the three surface profiles on each AFM image. The large standard deviation is expected for the measurements due to the fact that only one 30 × 30 μm image has been analyzed.

The value of strain where the initial deformation was observed corresponded well to in-situ XRD straining experiments as the strain, 1% strain, where the maximum stress was reached (Fig. 5b). This would indicate that the maximum stress (1050 MPa) most likely relates to the yield stress of the Cu film because of the observation of necking on the film’s surface. Yu and Spaepan [26] also observed similar yield stress values for Cu on PI using a similar technique. After the peak stress was reached, the measured stress in the film decreases with further straining. Upon unloading, the film stress transitions from tensile to compressive due to the elongation of the film and substrate. The Cu film elongates more than the underlying PET substrate and in order to accommodate the length, the film goes into a compressive state. The compressive state was also observed when the sample was removed from the straining stage and it curled with the film facing out, an indication of a film with a compressive stress. This transition from tensile to compressive stress is believed to aid in the recovery of resistance and will be discussed later.

**Fig. 6:**
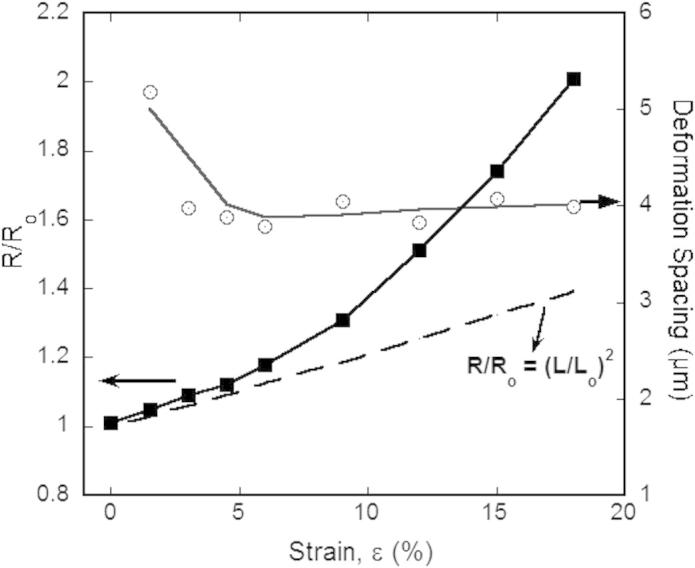
In-situ AFM-4PP results allow for the direct comparison of electrical resistance and deformation as a function of strain. The resistance (solid squares) does not start to deviate from the predicted theory until the first through thickness crack forms at approximately 5% strain. At this same strain, the deformation spacing (open circles) has also reached a saturation level.

The collected resistance data also increases with increasing strain. The original resistance, *R*_o_, of the film before straining was 0.545 Ω. As shown in Fig. 6, the resistance gradually increases, closely following the theoretical line of Eq. (3) until about 5% strain. At this point, the resistance starts to increase dramatically away from theory until reaching a relative resistance (*R*/*R*_o_) value of 2.01 at 18% strain. From the corresponding AFM images (Fig. 4) it is known that at the maximum strain, several through thickness cracks are the reason behind the 200% increase of resistance. Also from Fig. 6 the direct connection during straining between the measured electrical properties and mechanically induced damage in the form of deformation and cracking of the film can be seen. The deformation spacing reaches its saturation level when the first TTC form and when the resistance ratio deviates from theory.

From these experiments, it can be verified that deformation is occurring before a through thickness crack forms and that the deformation is accountable for the increase in the relative resistance ratio as well as the increase in probe spacing. If grain growth were occurring, the relative resistance would decrease [27]. This is demonstrated by the presence of localized necking between 1.5% and 6% strain. The small amount of deformation does not greatly affect the theoretical electrical behavior. However, as soon as a crack forms the electrical response will be altered. For this particular film-substrate combination, 5% strain could be considered as the critical strain for optimum electro-mechanical behavior.

**Fig. 7:**
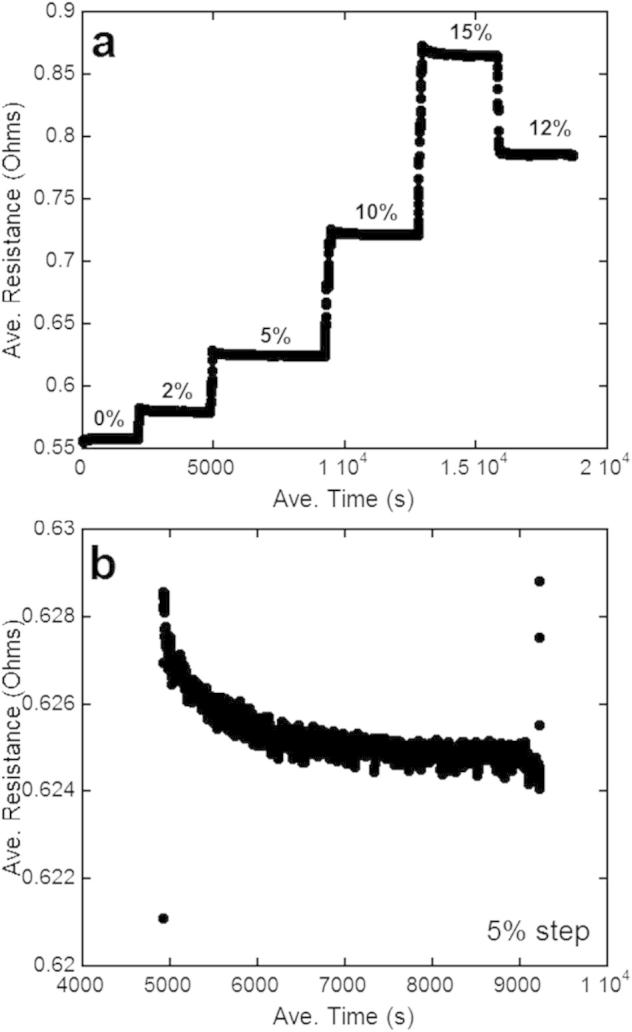
(a) Average measured resistance during AFM imaging. The plateaus correspond to different levels of applied strain (2–15%). (b) The average resistance data during the 5% applied strain step. Initially the resistance decreases, but after a few minutes, reaches a steady-state level. The difference between the resistance at the beginning of the hold and the end of the hold is about 1%, indicating that under load the resistance does not change significantly.

It should be noted that all of the AFM images and resistance values were taken while in the strained condition. Often when comparing results to those found in the literature, the resistance values were obtained in the *strained* condition, but the corresponding LM, SEM, or AFM image is of the *unstrained* condition. The experiment here presents resistance data and AFM images of the strained and unstrained condition. Images made after the sample is removed from the loading device can be misleading due to viscoelastic recovery of the substrates that occurs immediately after removal and causes crack closure [22]. It is known that with the experiment shown here, a similar effect occurs due to the long imaging times (approximately 15–30 min per straining step) required by the AFM. However, during the AFM imaging the resistance can be continuously recorded (Fig. 7a). Close examination of the resistance plateaus during AFM imaging reveal that the electrical resistance slightly decreases during the imaging hold, but after a few minutes, reaches a steady-state (Fig. 7b). The change in resistance is on the order of 0.005 Ω for each hold period and can be considered as insignificant (less than 1% change).

From the in-situ AFM-4PP experiment, the through thickness crack spacing was determined to increase when the sample is in an unloaded state and the resistance ratio reduced to 1.74 (Table 1). It should be mentioned that the deformation and crack spacing values have large standard deviations which overlap between the 18% maximum strained state and the 15% unloaded strain state. This arises due to the fact that only one 30 μm × 30 μm image can be analyzed for each straining step rather than a much larger area that can be viewed with SEM or LM. The resistance recovery observed with the increase of through thickness crack spacing is still an important result because it directly illustrates that crack closure occurs and can be quantified as an increase in the through thickness crack spacing (longer distances between through thickness cracks). Recall that the stress in the Cu film in the unloaded state was compressive (Fig. 6). The compressive state of the film also increases the probability that the cracks can close. This can be visualized by the fact that during straining the polymer substrate becomes elongated as the film deforms and fractures (also becoming elongated and increasing the resistance). Upon unloading, the deformed film is now longer than the polymer substrate. To accommodate the added length, the through thickness cracks close, reducing the resistance. Further work in this direction, to fully explain the relation between ductile deformation and electrical resistance as well as the resistance recovery would be necessary to completely understand the behavior.

## 4 Conclusions

A 200 nm Cu film on a 23 μm thick PET substrate was strained in-situ under the AFM and with 4PP electrical measurements. The goal of the combined in-situ fragmentation techniques was to try to understand how mechanically induced deformation and electrical properties were connected. With the AFM images, the through thickness crack spacing (only cracks) and the deformation spacing (cracks and localized necking) were quantified for each straining step up to 18% strain using the Δ*/h* ratio. The deformation and crack spacings were then compared to the measured film resistance at the same strains and increased from the expected theoretical values once the first through thickness crack formed in the film. In-situ XRD fragmentation was also employed to relate the stress present in the film to the observed damage (cracks and necks) during straining and unloading of the sample. The measured XRD stress-strain behavior correlated well to the observed deformation. The combined techniques of in-situ AFM-4PP fragmentation and the complementary in-situ XRD experiments help to shed light on the resistance recovery phenomenon and how it could be harnessed for future flexible electronic design.

## Figures and Tables

**Table 1:** Average deformation and through thickness crack spacing values and relative resistance ratio at 18% maximum strain and 15% unloaded strain.

Strain	Ave. deformation spacing (μm)	Ave. crack spacing (μm)	*R*/*R*_o_
18% (max)	4 ± 1.8	6.4 ± 3.3	2.01
15% (unload)	3.8 ± 1.6	8.1 ± 3.6	1.79
